# COVID-19 Pneumonia Complicated by Pneumomediastinum: A Case Report

**DOI:** 10.7759/cureus.32508

**Published:** 2022-12-14

**Authors:** João Cardoso, Ivo Castro, Vasco Gaspar, Cristina Esteves

**Affiliations:** 1 Department of Internal Medicine, Hospital de Santarém EPE, Santarém, PRT

**Keywords:** covid-19, emphysema subcutaneous, non-invasive mechanical ventilation, pneumonia, pneumomediastinum

## Abstract

Coronavirus disease 2019 (COVID-19) is a pandemic that spread rapidly around the world, causing an enormous overload on the health systems of the different affected countries. Among the many different manifestations of severe acute respiratory syndrome coronavirus 2 (SARS-CoV-2) infection, an uncommon complication is the development of pneumomediastinum.

In the clinical case presented, the patient was diagnosed with COVID-19 pneumonia and due to severe refractory hypoxemia, she was submitted to therapy with non-invasive ventilation (NIV). After initial stabilization and improvement, there was unexpected clinical deterioration and pneumomediastinum was diagnosed. The purpose of this report is to highlight the importance of considering pneumomediastinum as a complication of COVID-19 pneumonia in cases subjected to non-invasive ventilation.

## Introduction

Coronavirus disease 2019 (COVID-19) is a pandemic that originated in Wuhan, China, that spread rapidly around the world causing an enormous burden on the health systems of the different affected countries. Due to the limitation of available health resources it was necessary to adopt therapeutic strategies to respond to all critically ill patients admitted to hospitals and one was the use of non-invasive ventilation (NIV) in the treatment of severe COVID-19 pneumonia with respiratory failure not only as a bridging strategy to invasive mechanical ventilation (IMV) but also as therapeutic ceiling [1].

Among the many different manifestations of severe acute respiratory syndrome coronavirus 2 (SARS-CoV-2) infection, an uncommon complication of COVID-19 pneumonia is pneumomediastinum, which is characterized by the presence of free air in the mediastinum [2]. A structured survey of pneumomediastinum and its incidence was conducted in the United Kingdom among patients hospitalized with COVID-19 and found an incidence of 0.64% per inpatient admission (377 cases were identified from 58 484 inpatients with COVID-19) [3].

In the setting of COVID-19 pneumonia, pneumomediastinum is thought to be precipitated by sub-pleural alveolar rupture [2]. The pathophysiology of this condition remains obscure [4]. It could result directly from the pathogenesis of COVID-19 pneumonia or be a consequence of tracheal intubation or barotrauma from invasive and non-invasive ventilation [5]. Some authors related this association with the higher number of patients undergoing mechanical ventilation, however, a comparative analysis of ventilated patients showed a higher incidence in the group of COVID-19 patients, which may indicate that SARS-CoV-2 infection may have a causal role in pneumomediastinum [6].

Pneumomediastinum itself is mostly a self-limiting condition usually treated conservatively with the spontaneous resolution being the rule [1]. Albeit usually a benign situation, accumulation of air within the mediastinum can progress to tension pneumomediastinum with cardiovascular collapse [2].

## Case presentation

A 73-year-old female patient was admitted to the Emergency Department with a three days history of dyspnea, fever, and non-productive cough. She had a personal history of ischemic heart disease, hypothyroidism, obesity, arterial hypertension, and dyslipidemia. COVID-19 infection was suspected given the existence of infected family members. Upon physical examination, the respiratory rate was 30 cycles per minute, peripheral oxygen saturation (SpO2) 78% without supplemental oxygen, heart rate 106 beats/min, and blood pressure 88/42 mmHg. Pulmonary auscultation revealed bilateral basal crackles. High concentration oxygen mask (FiO2 85%) was placed with saturation improvement to 98%, and fluid therapy was performed with blood pressure normalization. Arterial blood gases under oxygen therapy and after fluid replacement showed pH 7.421, PaCO2 39.7 mmHg, PaO2 119 mmHg, lactate 1.1 mmol/L, and HCO3 25.6 mmol/L. With a target of SpO2 94%, oxygen therapy for the patient was reduced to FiO2 60% using a venturi mask.

A chest X-ray revealed multiple ground-glass opacities and bilateral hilar prominence. Laboratory data were significant for elevated inflammatory markers. The electrocardiogram showed sinus tachycardia. The patient was diagnosed with COVID-19 with a positive nasopharyngeal swab polymerase chain reaction for SARS-CoV-2. She started treatment with intravenous dexamethasone 6mg daily, inhaled 400µg twice daily, and empirical antibiotic therapy with intravenous ceftriaxone 2000mg daily and azithromycin 500mg.

On the third day of hospitalization (sixth day of symptoms), there was a clinical worsening with confusion, dyspnea, and hypoxemia to SpO2 80-82%. An evaluation was requested to the Intensive Care Unit (ICU) team and they decided that invasive mechanical ventilation was not in the patient’s best interests given the patient's frailty. Also, contributing to this decision was the fact that Portugal was at the peak of the second wave of COVID-19 and the available intensive care beds were scarce. High-flow nasal cannula (HFNC) therapy was not available at the hospital so, despite presenting with confusion, she was started on NIV (continuous positive airway pressure (CPAP) (10cmH2O, FiO2 60%) with a good response with SpO2 95% and observed tidal volume of 450-550mL. 

On the eighth day of hospitalization (11th day of symptoms), the hypoxemia worsened again with an increase in inflammatory parameters. It was necessary to adjust CPAP to 12cmH2O and FiO2 100%. Chest X-Ray was repeated and showed aggravation of multiple ground-glass opacities. Another request to accept the patient in the intensive care unit was made, but the decision to use NIV as a therapeutic ceiling was maintained. The decision was communicated to the patient and her family, who accepted the decision of the medical team. Antibiotic therapy was escalated to piperacillin + tazobactam 4.5g every six hours for suspected bacterial co-infection given the progressive rise in inflammation markers throughout hospitalization.

**Figure 1:**
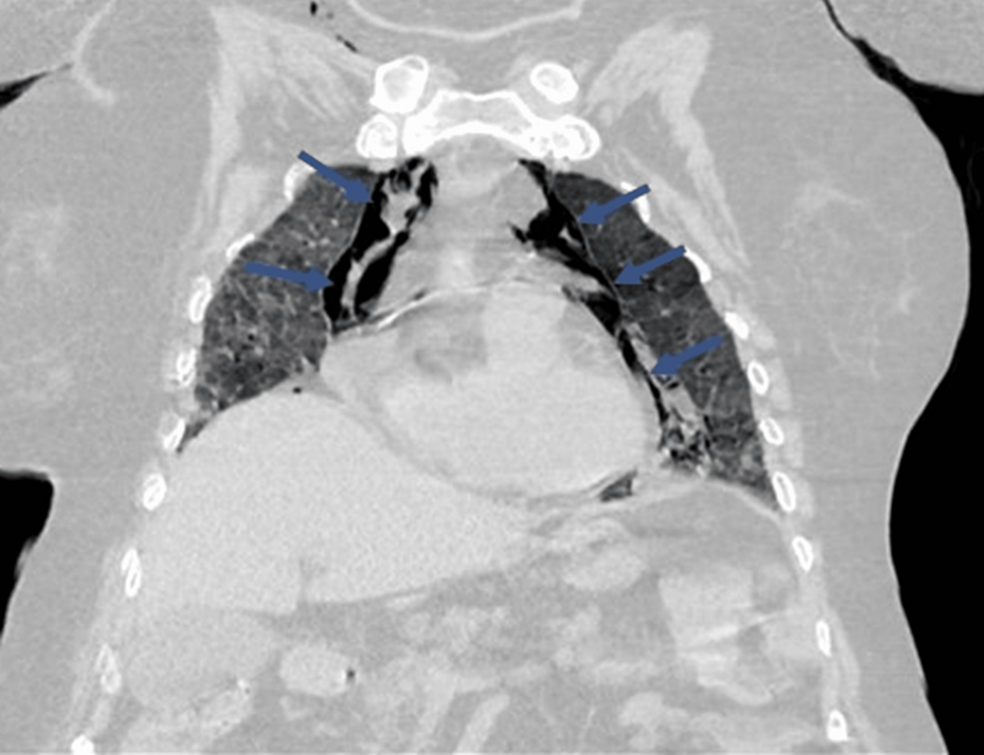
Computed tomography of the chest (coronal section) The image is revealing a large pneumomediastinum and cervical emphysema.

**Figure 2:**
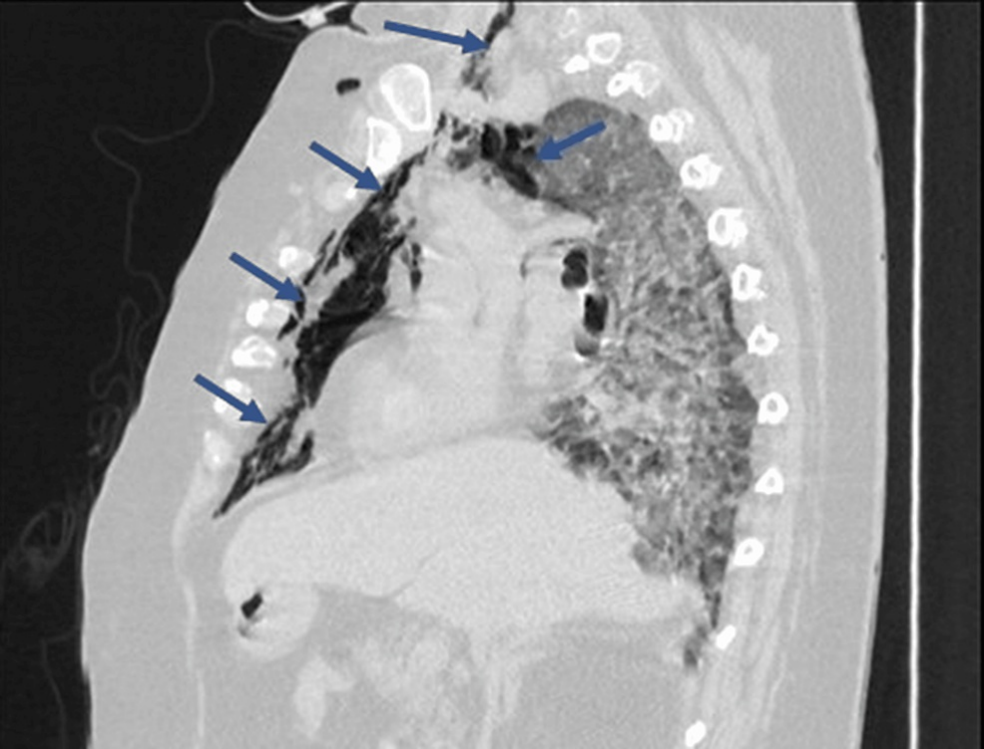
Computed tomography of the chest (sagittal section) Pneumomediastinum and subcutaneous emphysema are observed in this image.

The patient's condition was stable on the above NIV settings until the 10th day of hospitalization when another acute worsening occurred with desaturation to SpO2 85%. Arterial blood gases showed PaO2 54.5 mmHg (with FiO2 100%) and lactate 3.1 mmol/L. A chest computed tomography scan (Figures 1, 2) revealed a large pneumomediastinum extending to the right cervical region, extensive areas of ground-glass opacities, thickening of the interlobular septa and areas of consolidation involving all lung lobes bilaterally and diffusely. After the diagnosis of pneumomediastinum, the patient maintained NIV for another seven days with progressive worsening of hypoxemia. On the 17th day of hospitalization, a family visit was authorized and, together, it was decided to suspend NIV and initiate palliative care. The patient died on the 22nd day of hospitalization.

## Discussion

The increased reports of pneumomediastinum in patients hospitalized due to infection of SARS-CoV-2 during the COVID-19 pandemic are regenerating awareness of this infrequent diagnosis [2]. There seems to be an association between its appearance and more severe forms of the disease, in both ventilated and non-ventilated patients [5].

Pneumomediastinum due to barotrauma has been reported as a complication of NIV. However, before the widespread use of NIV in severe COVID-19, reports of pneumomediastinum in patients were very rare [1]. Recent studies have reported that mechanically ventilated patients with COVID-19 pneumonia have a higher incidence of developing pneumomediastinum than mechanically ventilated patients with acute respiratory distress syndrome (13.6% vs. 1.9%) suggesting that coronavirus infection may have a causal role [6]. Also, there are described subsets of patients developing pneumomediastinum without exposure to mechanical ventilation [2]. This could be explained by considering that in patients with COVID-19 infection, there is a combination of factors that include increased susceptibility of alveoli to rupture as well as increased stress applied to the respiratory system resulting from recurrent cough and increased work of breathing [2]. It is suggested to be more accurate to associate the development of pneumomediastinum in COVID-19 patients to the “lung frailty” caused by the underlying disease process of SARS-CoV-2 infection as opposed to barotrauma [6].

Another study, a structured survey of pneumomediastinum and its incidence in COVID-19 patients conducted in the United Kingdom demonstrates an incidence of pneumomediastinum in COVID-19 of 0.64% per inpatient admission. The incidence of spontaneous pneumomediastinum (without any mechanical ventilation), in this cohort, was 0.13%, much higher than the estimated background rates of non-COVID-19 spontaneous pneumomediastinum of 0.00002% [3]. In this study, the largest reported series of pneumomediastinum in COVID-19 disease, pneumomediastinum appears to be a marker of severe pneumonitis, and not necessarily as a result of the use of positive pressure ventilation (CPAP or invasive ventilation). However, it may also indicate an important role of mechanical ventilation in the development of pneumomediastinum in COVID-19 pneumonia [3]. Also, there was no evidence of increased harm caused by continuing CPAP in COVID-19 patients who developed pneumomediastinum [3]. 

The causes of pneumomediastinum in mechanically ventilated patients can be multifactorial, and the fact is that despite the criteria of protective ventilation being respected, in COVID-19 patients submitted to invasive ventilation, different pressures like positive end-expiratory pressure, peak airway pressure, and plateau airway pressure were higher compared to patients without COVID-19 pneumonia [6].

Also, the estimate of incidence is also subject to diagnostic biases as the main mode of diagnosis of pneumomediastinum is computed tomography (CT) scan and many were pulmonary angiogram studies assaying for pulmonary emboli, not for pneumomediastinum. For 46.2% of the patients diagnosed with pneumomediastinum on thoracic CT, the pneumomediastinum was not visible on their preceding chest radiograph so there are likely to be a number of undetected cases [3].

In the clinical case presented, the patient was treated in a secondary hospital in Portugal at the peak of the second wave of COVID-19, the worst in January 2021. The available resources were scarce, both human and material resources, and supply shortages of medical resources were even more exacerbated by disruptions to the global supply chain [7]. High-flow nasal cannula therapy was not available at the hospital and therapy with NIV was realized in wards. Also, healthcare workers faced numerous physical and mental challenges due to increased workloads, constant exposure to infection, and burnout [7], which may have impacted patient management during the COVID-19 pandemic and predisposed COVID-19 patients to remain in patient-ventilator asynchrony leading to unfavorable airway pressures and barotrauma.

## Conclusions

Pneumomediastinum is an uncommon complication of COVID-19 pneumonia that appears to be a marker of severe pneumonitis, in both ventilated and non-ventilated patients. Given the high number of patients with COVID-19 pneumonia undergoing respiratory support with NIV, it is mandatory to consider the development of pneumomediastinum if there is unexpected clinical deterioration. Diagnosis by chest X-ray is difficult, often requiring computed tomography. We want to highlight the importance of considering pneumomediastinum as a complication of COVID-19 pneumonia in cases of NIV refractoriness.
